# Preclinical evaluation of [^68^Ga]Ga-AAZTA-FAPI-46: a novel PET tracer for targeting fibroblast activation protein (FAP)

**DOI:** 10.1186/s41181-025-00375-2

**Published:** 2025-08-05

**Authors:** Rebecca Rizzo, Paolo Rainone, Rachele Stefania, Sara Belloli, Silvia Valtorta, Angela Coliva, Marco Maspero, Lidia Avalle, Martina Capozza, Rosa Maria Moresco, Calogero D’Alessandria, Enzo Terreno

**Affiliations:** 1 Center for Biotechnology and Translational Medicine, University of Turin, Piazza Nizza 44/bis, Turin, 10126 Italy; 2 Department of Nuclear Medicine, TUM University Hospital rechts der Isar, 81675 Munich, Germany; 3 DISIT, University of Eastern Piedmont, Viale Teresa Michel 11, Alessandria, 15121 Italy; 4 Nuclear Medicine and PET Cyclotron Unit, IRCCS Ospedale San Raffaele, Milano, Italy; 5 Institute of Molecular Bioimaging and Physiology-IBFM, CNR, Segrate, Italy

**Keywords:** AAZTA, FAP, PET, FAPI-46, GALLIUM-68

## Abstract

**Background:**

The aim of this work was to demonstrate the suitability of AAZTA chelator conjugated to a FAPI-46-derived FAP inhibitor and labelled with gallium-68 as a potential PET tracer.

**Results:**

Gallium-68 radiolabelling was achieved with high radiochemical yield at room temperature. The new tracer was stable in different media, showing specific binding to FAP-protein both in vitro and in vivo, and a suitable biodistribution and clearance. High tumor uptake of the tracer (1.01 ± 0.12 SUV 35 min p.i.) was found in 4T1-tumor bearing mice, and blocking experiments demonstrated the high target specificity.

**Conclusion:**

The substitution of the DOTA chelator with the AAZTA ligand on FAPI-46 moiety allowed a fast radiolabelling at room temperature of the PET tracer without influencing the biodistribution properties, such as clearance and FAP-mediated tumor uptake, but rather expanding the tracer applicability.

**Supplementary Information:**

The online version contains supplementary material available at 10.1186/s41181-025-00375-2.

## Introduction

In the era of radio-theranostics, the AAZTA chelator has been attracting increasing interest thanks to its ability to complex diagnostic and therapeutic trivalent radiometals (such as ^68^Ga, ^44^Sc and ^177^Lu) using mild labeling conditions (Nagy et al. 2017; Sinnes et al. 2020). In fact, the most widely used chelators for radiometals, i.e. macrocyclic derivatives based on DOTA and NOTA chelators, typically require high or moderate temperatures to achieve high labelling yields. Moreover, for theranostic strategies involving ^177^Lu, only DOTA can be used, requiring labelling at temperatures close to 100 °C, condition at which many molecular targeting vectors (e.g., those based on proteins) are unstable. Among a panel of candidates for room temperature radiolabeling (Tsionou et al. 2017) (e.g., HBED: N,N’-bis(2-hydroxybenzyl)ethylenediamine-N, N’-diacetic acid (Klika et al. 2021), THP: tris(hydroxypyridinone)(Young et al. 2017), and DATA: 6-amino-1,4-diazapine-triacetate) (Sinnes et al. 2019), AAZTA was reported to successfully chelate not only Ga-68 but also Lu-177 and Sc-44, making it suitable for theranostic applications. AAZTA is an heptadentate aminopolycarboxylate ligand with a 1,4-diazepine scaffold that has been extensively studied as chelator of Gd^3+^ ions for MRI applications (Gugliotta et al. 2009; Aime et al. 2004). This ligand has already been proven to form thermodynamically stable and kinetically inert Ga^3+^ complexes (Baranyai et al. 2013), and several AAZTA conjugates were developed over the last years. As examples, Manzoni et al. demonstrated the formation of ^68^Ga-AAZTA-RGD complex at room temperature in acetate buffer at pH 3.8 in 10 min (Manzoni et al. 2012), and CyAAZTA was successfully radiolabelled with gallium-68 in acetate buffer at pH 3.8, in 15 min at room temperature by Vagner and coworkers (Vágner et al. 2016). In the field of theranostics, over the last 10 years the pan-cancer biomarker Fibroblast Activation Protein (FAP) has gained increasing attention (Calais 2020). FAP, known also as seprase (O’Brien and O’Connor 2008), is a transmembrane type-II protein belonging to the family of dipeptidyl peptidases (Levy et al. 1999). FAP has been recently attracting interest since its involvement in angiogenesis, growth, aggressiveness, progression, and prognosis of cancer (Hamson et al. 2014). Low basal levels of FAP expression might be found in many tissues, including the bone marrow, adipose, and skin (Fitzgerald and Weiner 2020). Being involved in the extra-cellular matrix (ECM) tissue remodeling processes (Bremnes et al. 2011), FAP was found to be present also in such non-cancer disease like fibrosis (Williams et al. 2015), arthritis (Wang et al. 2023), atherosclerosis (Waumans et al. 2015) and infarction (Borne et al. 2010). But more importantly, low levels of FAP were found in healthy tissues (Hua et al. 2011), whereas it is overexpressed by CAFs (cancer associated fibroblasts) in more than 90% of epithelial tumors (Costa et al. 2018), including breast, lung, pancreas, brain and colon cancer (Kratochwil et al. 2019). Since the development of the most potent FAP inhibitor named UAMC1110 (Decker et al. 2019), several radiopharmaceuticals have been developed for PET, SPECT and therapy purposes (Altmann et al. 2021; Xin et al. 2021; Kuyumcu et al. 2021). Remarkable examples include FAPI-04 (Lindner et al. 2018) and FAPI-46 (Loktev et al. 2019) ligands. This work mainly aims at synthetizing a new AAZTA-based FAP inhibitor using the FAPI-46 structure and optimizing the room temperature radiolabeling with gallium-68 for PET purposes (Moon et al. 2020, 2021). Moreover, the tracer was characterized in terms of (radio)stability in different media, affinity towards FAP expressing cells and in vivo performances as potential PET tracer.

## Methods

### Materials

All chemicals were purchased from Sigma-Aldrich, except for the 6-bromoquinoline-4-carboxylic acid that was purchased from Biosynth^®^Carbosynth. All the solvents were purchased from VWR. Analytical UPLC-MS were acquired by Waters Acquity^®^ UPLC equipped with TUV and QDa detectors on a BEH C18 column (1.7 μm, 2.1 × 50 mm) equipped with Acquity^®^ UPLC BEH C18 VanGuard™Pre-column (300Å, 1.7 μm, 2.1 × 5 mm). Preparative HPLC-MS were carried out on a Waters FractionLynx autopurification system equipped with Waters 2996 diode array and Micromass ZQ (ESCI ionization mode) detectors on an Atlantis Prep dC18 OBD™ (5 μm, 19 × 150 mm) column. ^1^H-NMR spectrum of AAZTA-FAPI-46 were measured on a Bruker Avance spectrometer (400 MHz) instrument. Chemical shifts are reported in parts per million (ppm) and are referenced to tetramethylsilane. Radio-HPLC quality controls on radiolabeled AAZTA-FAPI-46 were performed on a Waters HPLC-MS system equipped with a Waters 1525 binary pump. Gel 60 F_254_ strips (Merck KGaA, Darmstadt, Deutschland) and ITLC-SG strips (Agilent Technologies, Didcot, UK) were used for radio-TLC quality controls. Sep-Pak C18 Light Cartridges (Waters) were used to purify the crude of reaction.

### Synthesis of AAZTA-FAPI-46 chelator

The synthesis of the AAZTA-FAPI-46 was partially based on already published procedure (Loktev et al. 2019). The amide coupling between the simil-FAPI-46 precursor and the AAZTA^5^ chelator was performed through a standard amide coupling. See Supplementary Information for synthesis, purification procedures, UPLC-MS and NMR spectra (Fig. S2, S3).

### Gallium-68 manual radiolabelling

Elution of gallium-68 was performed with 0.1 M hydrochloric acid from a ^68^Ge/^68^Ga generator (Scintomics, Germany) in a volume of 1-1.2 mL containing the maximum activity of 410–460 MBq. Preliminary tests were conducted by using ca. 20 MBq to achieve the optimal experimental conditions. Aqueous solutions of HEPES 0.4 M or sodium acetate 0.4 M were used to adjust the pH of reaction. The reactions were conducted at room temperature.

Gallium-68 labelling of AAZTA-FAPI-46 was optimized by investigating the influence of pH, precursor concentration and time of reaction. Firstly, the optimal value of pH was assessed by using the precursor concentration of 40 µM (6.6 nmol). The different pH values and buffer conditions tested are listed in the Supplementary Information.

After finding the optimal pH value relying on the maximum % labelling of gallium-68, different AAZTA-FAPI-46 precursor concentrations were tested (0.5, 1, 5, 10, 15, 20, 40, 70, 100 µM). Finally, the kinetic of reaction was studied and aliquots were taken at 1, 3, 5, 10 and 15 min to measure the radiochemical yield.

Quality controls were performed by radio-TLC measurements using sodium citrate 1 M pH = 5 (considering the following retention factors (Rf): [[^68^Ga]Ga-AAZTA-FAPI-46)] Rf < 0.1; ^68^Ga colloids: < 0.1; soluble ^68^Ga^3+^ Rf > 0.9) and Silica Gel 60 F_254_ strips and by radio-HPLC measurements. Purification was performed prior to in vitro experiments. The crude solution was worked-up by solid-phase extraction. After reaction time, the solution was diluted into 8 ml of water and then load bottom-up on the cartridge. The resulting syringe solution was the waste solution ^68^Ga-AAZTA-FAPI-46 was eluted from the cartridge with 500 µL of water/ethanol 1:1 solution. Radiochemical purity (RCP%) was assessed by radio-HPLC measurements.

### Gallium-68 automated radiolabelling

The preparation of [^68^Ga]Ga-AAZTA-FAPI-46 was automated on a GallElut^+^ system (Scintomics, Germany). Briefly, the ^68^Ge/^68^Ga generator eluate was buffered to pH = 3.5 using HEPES 2.7 M. AAZTA-FAPI-46 was then added to the gallium-68 solution (410–460 MBq, ca. 1.2 mL). The mixture was incubated at room temperature for 10 min before passing through a SPE cartridge (Waters, tC18 environmental cartridge), previously conditioning by purging with absolute ethanol (5 mL, Ph. Eur.) and water (10 mL, Ultrapure Water). The cartridge was then purged with water (10 mL) and air (10 mL). The labelled product was eluted from the cartridge into a 10 mL flask with a 1:1 mixture of ethanol and water (2 mL), following by purging with PBS buffer (1 mL, pH = 7.4) and water (1 mL). The production time was approximately 30 min.

### Stability studies

Stability studies of [^68^Ga]Ga-AAZTA-FAPI-46 were performed in human serum, PBS and EDTA 5 mM over a period of 2 h at 37 °C. Briefly, the purified compound obtained with a radiochemical purity greater than 98%, was used for the stability studies by diluting in a ratio of 1:5 (400 µL of human serum, PBS or EDTA 5mM + 100 µL of labelled compound). For stability studies in serum, proteins were precipitated by adding acetonitrile. After the incubation time (0.5 h, 1 h, 2 h) the sample vial was centrifuged for 5 min at 9000 rpm, 50 µL of the supernatant was collected and analysed by radio-HPLC. For radio-HPLC detail procedure see Supplementary Information.

### Lipophilicity measurements

Lipophilicity (expressed by the logD_7.4_ value) was determined via the “shake-flask” method. Briefly, after the purification step, the pH of the product solution was adjusted to 7.4 with NaOH 1 M. 2–3 MBq of product were added to a 1 ml of a solution of 1:1 PBS (pH 7.4) and octanol mixture (*v*/*v*). The vials were vortexed for 3 min, and then centrifuged for 5 min at 13,000 rpm. The water and octanol fractions were separated, and the activity measured with a γ-counter (PerkinElmer, Beaconsfield, UK).

### Cell cultures and in vitro experiments

HEK293-FAP cells, stably expressing FAP were generated by Dr. Lidia Avalle. pCMV6-Kan/Neo-mFAP expression vector encoding murine FAP was purchased from Origene (cat. MC206606) and used to transfect HEK293 cells by means of Lipofectamine (Invitrogen, cat. 11668019). Transfected cells were then selected with 500ug/ml Geneticin (G-418, Gibco) for 7 days. In parallel, HEK293 cells receiving a construct without FAP coding sequence were generated as negative controls. FAP expression was measured by means of Western Blot.

For radioligand binding studies, HEK293T-FAP expressing cells (3 × 10^5^ /well) were plated on 96-well plates 24 h prior to the experiment. On the day of the experiment, media was removed, and cells were washed with ice-cold PBS. Competition experiments were performed by simultaneous exposure to unlabelled compound (10^− 11^ to 10^− 4^ M) and [^68^Ga]Ga-AAZTA-FAPI-46 (0.5 µM) for 1 h at 4 °C. After 1 h incubation, the supernatant was collected, the cells were rinsed twice with ice-cold PBS, lysed with NaOH 1 M for 10 min and bound fraction collected into vials. The experiment was performed five times. The radioactivity was assessed using a γ-counter (PerkinElmer, Beaconsfield, UK). For data treatment, the bound fraction (cells pellet) activity was divided to the total activity (bound + supernatant) and the results expressed as binding %.For internalization experiments, HEK293T-FAP expressing cells were incubated for 1 h at 37 °C with of [^68^Ga]Ga-DOTA-FAPI-46. After 20, 40 and 60 min of incubation, media and subsequent PBS washings were discarded, and the surface-bound radioactivity was removed by washing the cells with 400 µL of ice-cold 50 mM glycine in 150 mM NaCl (pH = 3) for 5 min on ice, followed by two washing with ice-cold PBS. To obtain the cell internalized fraction, cells were lysed with 400 µL of 1 M NaOH. The radioactivity in both fractions was measured by a γ-counter.

### In vivo studies

#### Cell culture

The 4T1 murine cells, which exhibit characteristics consistent with a triple-negative breast cancer (TNBC) phenotype, were originally derived from BALB/c mice. These 4T1 cells (sourced from ATCC) were maintained in RPMI 1640 medium with 10% foetal bovine serum (FBS), alongside 100 units/mL of penicillin and 100 µg/mL of streptomycin (GIBCO, Life Technologies, Monza, MB, Italy). Cultures were incubated at 37 °C in a humidified environment containing 5% CO₂. The medium was refreshed every 2–3 days, and cells were harvested using 0.25% trypsin/EDTA solution (GIBCO, Life Technologies, Monza, MB, Italy). Regular screening for Mycoplasma contamination was conducted using a MycoAlert detection kit (BioWhittaker-Lonza, Euroclone S.p.a., Milan, Italy).

#### Animal studies

Animal studies were conducted following institutional protocols for the care and use of experimental animals, which were approved by the Italian Ministry of Health (authorization n° 38/2024-PR). The animals were kept in a controlled environment with a constant temperature of 23 °C, 40% relative humidity, and maintained on a regular light/dark cycle. Food and water were available *ad libitum*. Seven to eight-week-old male Balb/c mice, obtained from ENVIGO RMS s.r.l., Italy, were subcutaneously injected with 5 × 10⁵ 4T1 breast cancer (BC) cells suspended in PBS (total volume 200 µl) in the right flank. Tumour growth was periodically monitored using callipers, and tumour volume calculated using the formula: (l² * L)/2, where “l” represents the smaller side, and “L” the larger one. Upon reaching an average tumour volume of 307 ± 67.4 mm³, approximately 15 days post-tumor cells subcutaneous inoculation, in vivo PET/CT imaging and biodistribution studies were carried out.

For kinetic analysis, [^68^Ga]Ga-AAZTA-FAPI46 or [^68^Ga]Ga-DOTA-FAPI46 (≈ 2 MBq/mouse in 100 µl of saline solution with < 10% ethanol) was administered intravenously via the tail vein to 4T1 tumour-bearing mice (*n* = 4 per radiotracer). The tumour uptake was monitored by whole-body PET/CT scans using preclinical β-cube^®^ and X-cube^®^ scanners (Molecubes, Gent, Belgium) performed at 30, 90, and 150 min post-injection (acquisition time 20 min. each). During imaging, mice were placed side by side under anaesthesia (2% isoflurane in medical air).

For inhibition studies, mice received an intravenous injection of an excess of the unlabelled AAZTA-FAPI46 precursor (50 µg in saline) 10 min before the injection of the [^68^Ga]Ga-AAZTA-FAPI46 (~ 2 MBq/mouse) (*n* = 5). The procedure was repeated after intravenous pre-injection of the vehicle solution on a second control group of mice (*n* = 5). After one hour, the mice underwent whole-body PET/CT acquisitions for 20 min and were then sacrificed for ex vivo quantification of radiotracer uptake. Mice were euthanized by cervical dislocation, and selected organs and tumours were collected, rinsed, weighed, and analysed for radioactivity content using a gamma counter.

CT and PET images were reconstructed using proprietary Molecubes software included with the system. CT images were reconstructed with a 200 μm isotropic pixel size using the ISRA algorithm. PET images were reconstructed with the List-Mode OSEM algorithm with 30 iterations and a 400 μm isotropic voxel size, including tracer decay correction, for the kinetic study the first acquisition (at 30 min p.i.) was reconstructed in two different and consecutive frames of 10 min each. CT/PET images were further processed through Region of Interest (ROI) analysis using PMOD software v.4.1 (Zurich, Switzerland). Radioactivity uptake was expressed as the standardized uptake value (SUV), calculated by the formula: tumour concentration activity [MBq/g]/(injected activity [MBq]/animal weight [g]). SUV mean values was employed for both kinetic and blocking studies, and results were also expressed as tumour-to-muscle ratio (T/M). For ex-vivo biodistribution, the radioactivity concentration was reported as SUV, and results also presented as tumour-to-muscle ratio (T/M).

### Statistical analysis

The Student’s t-test was used to compare the differences between the two groups. A p-value of less than 0.05 was considered statistically significant. Graph preparation and statistical analysis were performed using GraphPad Prism version 8.

## Results

### Radiolabelling

**Fig. 1:**
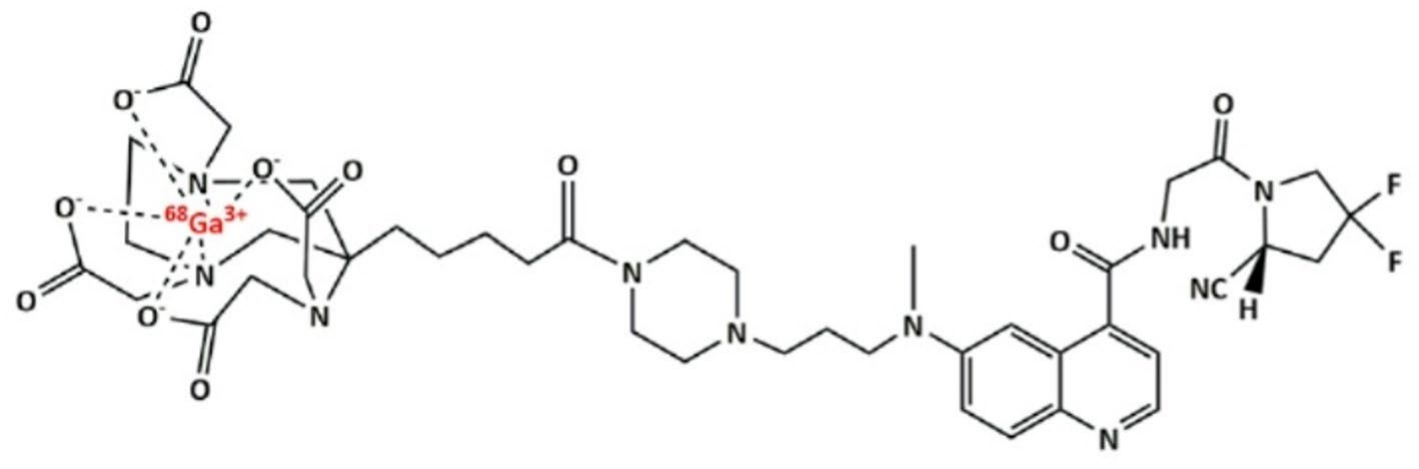
[^68^Ga]Ga-AAZTA-FAPI46 chemical structure.

**Fig. 2:**
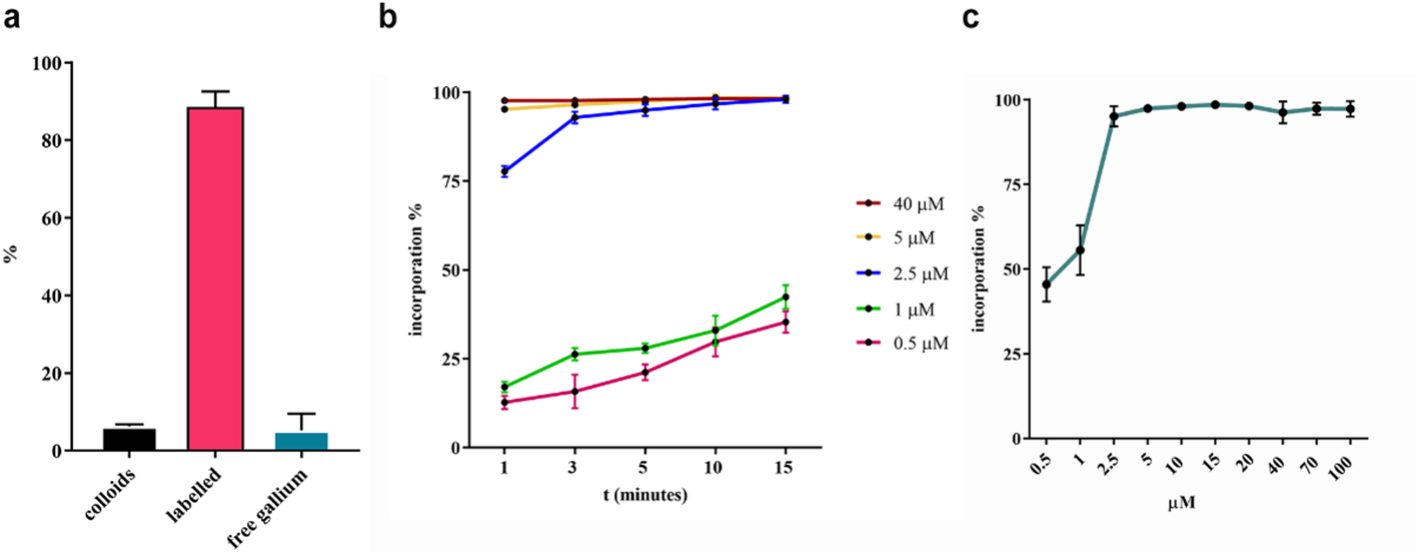
(**a**) Quantification of free gallium, colloidal species and Ga-complex 15 min after the addition of ≈ 20MBq of Ga-68 to a solution of AAZTA-FAPI-46 40 µM, pH = 3.5 at RT. (**b**) Kinetics of [^68^Ga]Ga-AAZTA-FAPI-46 at RT for tracer concentration ranging from 0.5 to 40 µM (RT, pH = 3.5). (**c**) Gallium-68 incorporation of AAZTA-FAPI-46 with different precursor concentration (RT, 15 min, pH = 3.5).

Gallium labelling of AAZTA-FAPI-46 (chemical structure reported in Fig. 1, synthetic procedure reported in Fig. S1) was first performed with a precursor concentration of 40 µM (6.6 nmol) and time of reaction of 15 min to assess the optimal pH value of reaction (Fig. S4). The reactions conducted at pH value of 3.5 led to the higher radiochemical yield, hence this condition was further tested in triplicate (Fig. 2). It was found that the labelling conducted ad pH = 3.5 led to a minimal colloidal formation as well as a minimal free gallium content. This result is in agreement with the Ga^3+^-AAZTA system speciation diagram obtained from pH-potentiometric titration by Baranyai and co-workers (Baranyai et al. 2013), from which it follows that in the 3–4 pH range there is the maximum abundance of Ga^3+^ for complexation with AAZTA chelator and a minimal formation of Ga(L)OH colloid. Different precursor concentrations were then tested, and an almost quantitative incorporation was achieved at 5 µM of precursor (Fig. 2A).

The complexation of AAZTA-FAPI-46 at room temperature with gallium-68 showed a very rapid kinetics achieving high incorporation already after 1 min (> 70%) for precursor concentration of 2.5 µM (0.385 nmol, Fig. 2B), confirming previous reported data (Sinnes et al. 2020; Ballal et al. 2020). For ligand concentration ≥ 5 µM, no substantial differences were observed, demonstrating an excellent gallium-68 complexation. An automated procedure allows a greater control over reaction times, temperatures and flow rates and may be quicker and more reproducible than the manual approach. The optimization of the automated synthesis protocol was performed by measuring the resulting activity into the product vessel, the waste vessel and the SPE cartridge. Radio-TLC and radio-HPLC measurements were done to evaluate the efficiency and the reproducibility of the process. The radiochemical yield was 84.97 ± 1.37%.

### Stability studies

**Fig. 3:**
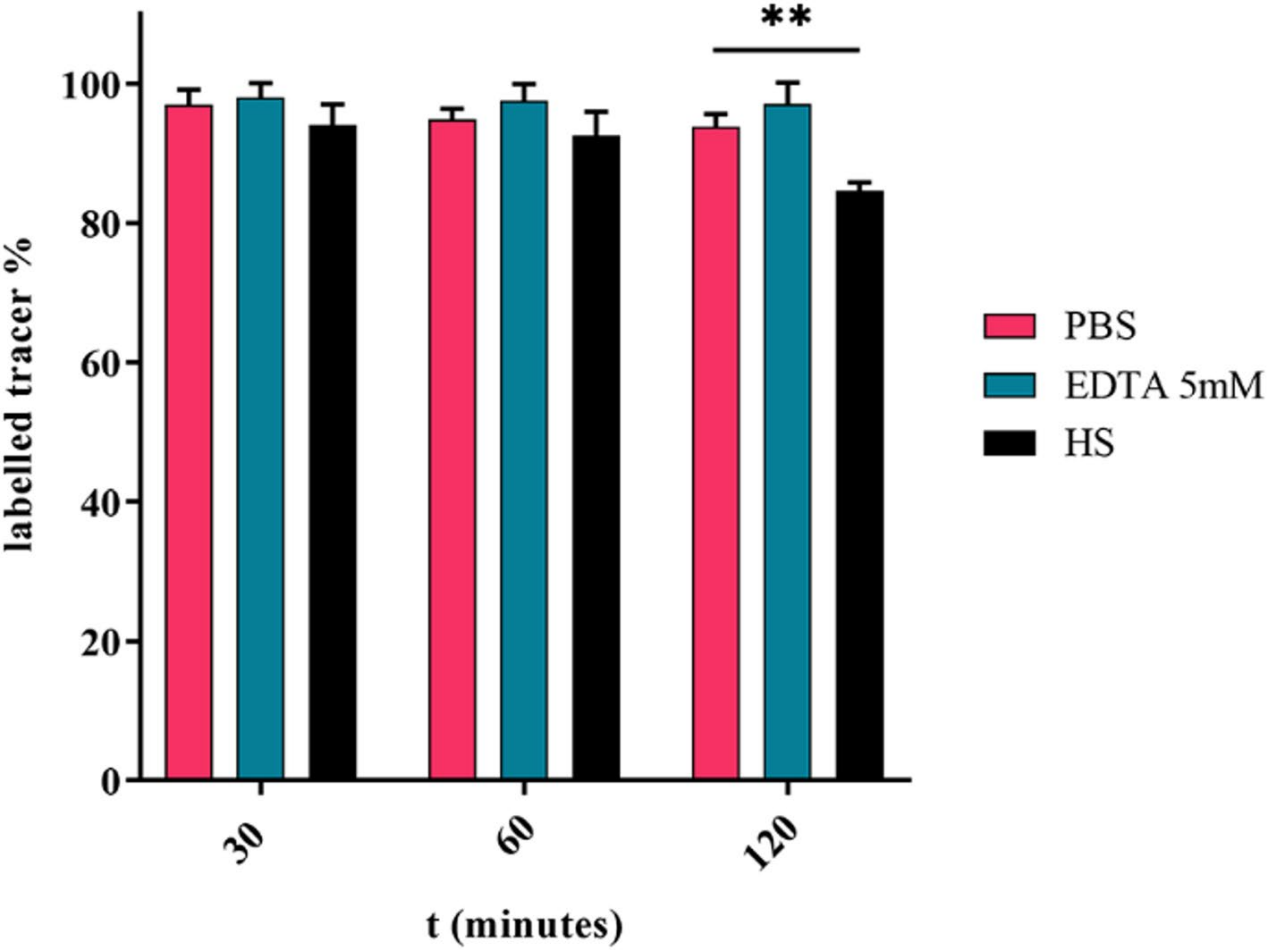
Stability investigation of [^68^Ga]Ga-AAZTA-FAPI-46 in HS, PBS and EDTA over 2 h at 37 °C. (*t*-test, p**=0.0012)

The stability of [^68^Ga]Ga-AAZTA-FAPI-46 in different media was investigated at 37 °C for 2 h. Figure 3 reports the percentage of intact compound after 30, 60 and 120 min of incubation. [^68^Ga]Ga-AAZTA-FAPI-46 proved to remain almost intact both in PBS and EDTA solutions, and no transchelation reaction was observed over 120 min. The excellent stability in vitro was also demonstrated in human serum, where a slightly reduction of the labelled compound (down to 84%) was observed at 120 min only.

### Lipophilicity measurements

The logD_7.4_ value of FAPI-04, which contains the DOTA chelator for gallium labelling, is reported in the literature as − 2.4 ± 0.28, confirming the hydrophilic character of ^68^Ga-DOTA complexes. Although the [^68^Ga]Ga-AAZTA-FAPI-46 net charge was − 1 in comparison with the neutral charge of the DOTA analogue, the obtained value of the logD_7.4_ of the [^68^Ga]Ga-AAZTA-FAPI-46 was − 2.48 ± 0.07, thus suggesting a minimal role of the chelators on the lipophilicity of the FAPi radiopharmaceuticals.

### In vitro cell binding studies

**Fig. 4:**
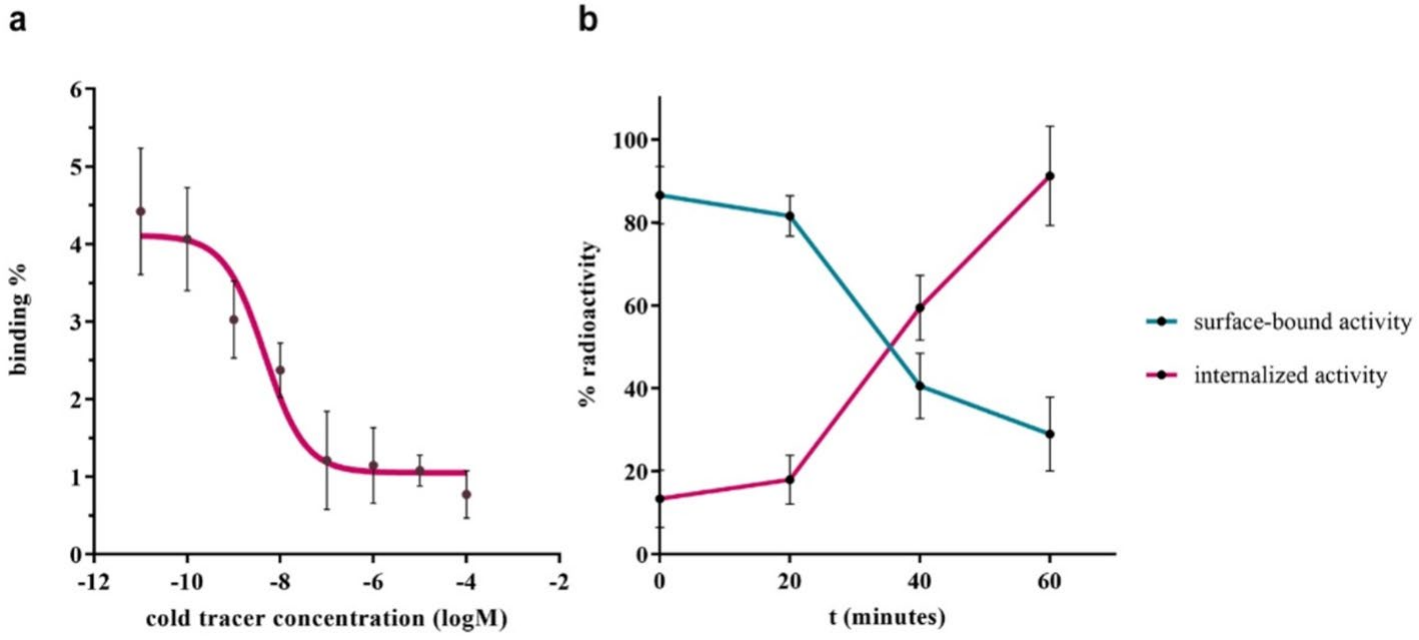
(**a**) Competition experiment on HEK293T-FAP cells at 4 °C for 1 h ([^68^Ga]Ga-AAZTA-FAPI-46 vs. unlabelled tracer). (**b**) Internalization experiment on HEK293T-FAP cells at 37 °C for 1 h.

To confirm the ability to target FAP, the [^68^Ga]Ga-AAZTA-FAPI-46 agent was incubated with HEK293T-FAP cells for uptake and internalization experiments. The substitution with the AAZTA chelator did not influence the binding affinity to FAP-expressing cells, as shown from the potency value in the nanomolar range (IC_50_ = 4.61 ± 0.21 nM), obtained by fitting the competition assay data with a nonlinear regression algorithm (GraphPad Prism 8 Software), which is consistent with previously published data for [^68^Ga]Ga-DOTA-FAPI-46 (13.5 nM) and other similar radiolabelled FAPI agents (Lindner et al. 2018; Loktev et al. 2019). Even the cell uptake results (Fig. 4b) were comparable with data reported for [^68^Ga]Ga-DOTA-FAPI-46, which showed an internalized fraction of 97.18% after 1 h of incubation in HT-1080-FAP cells (Loktev et al. 2019).

### In vivo PET imaging and ex vivo biodistribution

**Fig. 5:**
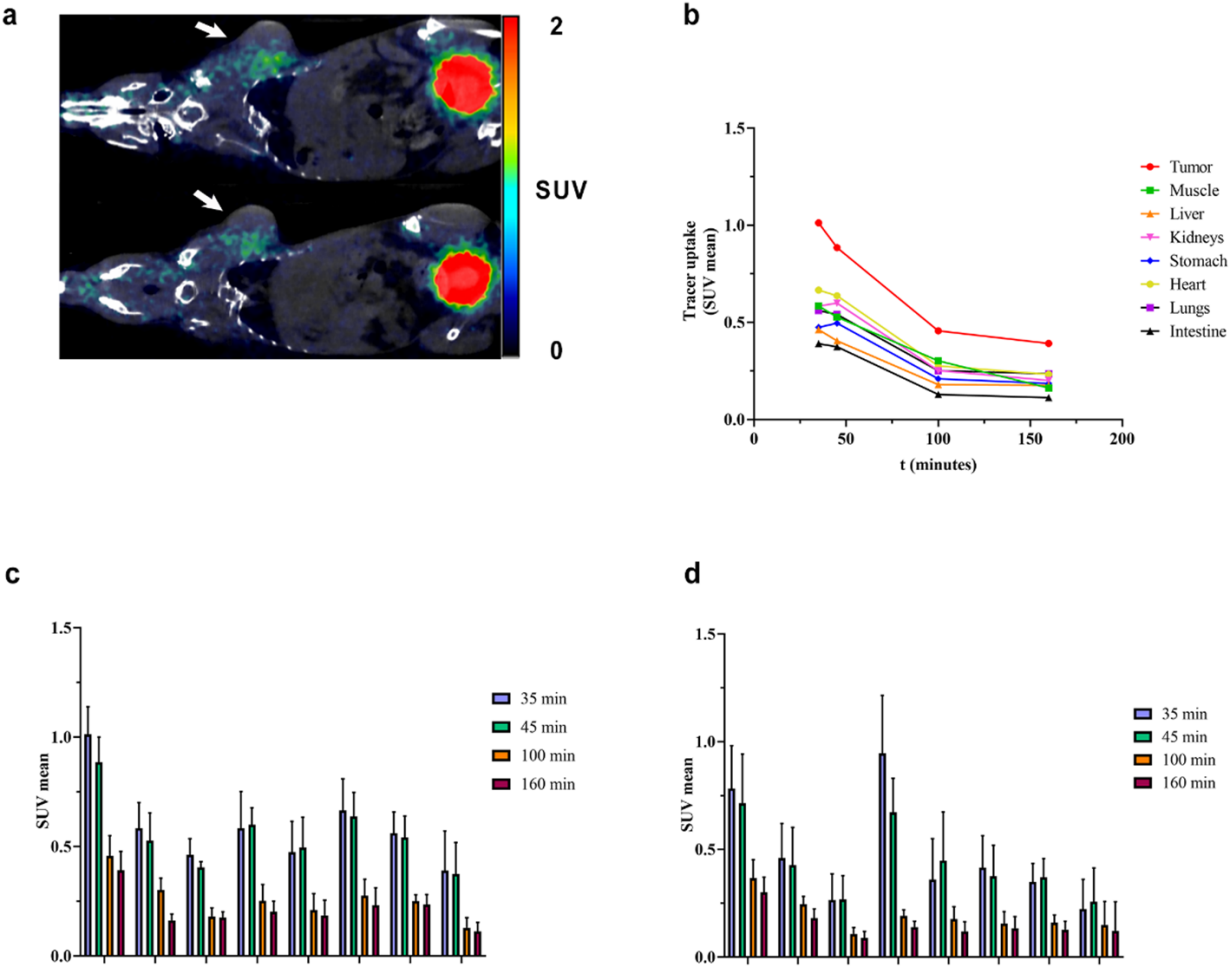
(**a**) PET/CT images acquired 45 min p.i. of ≈ 2.2 MBq of [^68^Ga]Ga-AAZTA-FAPI-46 (on the top) or [^68^Ga]Ga-DOTA-FAPI-46 (on the bottom) in 4T1-tumor-bearing mice. White arrows indicate tumors. Acquisition time: 20 min. PET and CT images are reported in Fig. S6. (**b**) Kinetic curves for [^68^Ga]Ga-AAZTA-FAPI-46 in 4T1 tumor–bearing mice determined by small-animal PET imaging in the main organs. (**c**) Organ SUV_mean_ of [^68^Ga]Ga-AAZTA-FAPI-46 in 4T1 tumor–bearing mice determined by small-animal PET imaging (*n* = 8). (**d**) Organ SUV_mean_ of [^68^Ga]Ga-DOTA-FAPI-46 in 4T1 tumor–bearing mice determined by small-animal PET imaging (*n* = 8)

Small animal PET/CT imaging of [^68^Ga]Ga-AAZTA-FAPI-46 on subcutaneous 4T1-tumor mice model was conducted, using [^68^Ga]Ga-DOTA-FAPI-46 as control group (**Fig. 5a** - for PET and CT images, refer to Fig. S6). The semi-quantitative analyses reported in Fig. 5c and d for [^68^Ga]Ga-AAZTA-FAPI-46 and [^68^Ga]Ga-DOTA-FAPI-46, respectively, reveals a comparable organs distribution kinetics in the examined timeframes. In particular, data indicated a rapid tracer accumulation in tumour at 35 min p.i., with a subsequent decrease. A similar trend was observed in the other peripheral districts, although with lower uptake values (Fig. 5b). Notably, in comparison with [^68^Ga]Ga-DOTA-FAPI-46, [^68^Ga]Ga-AAZTA-FAPI-46 reached higher SUV mean values in tumour (1.01 ± 0.12 vs. 0.78 ± 0.19 SUV mean; *p = 0.0192 at 35 min p.i.) and in the other tissues except in muscle, stomach and intestine. Conversely, in kidney the [^68^Ga]Ga-DOTA-FAPI-46 showed significant higher levels of uptake at 35 min p.i. (*p = 0.0110). Results expressed as tissue/background (muscle) ratios (Fig. S5) confirmed these findings and revealed, at 160 min p.i., a significant increase (*p = 0.0353) of the [^68^Ga]Ga-AAZTA-FAPI-46 levels in tumour (2.45 ± 0.59, tissue/muscle ratio) compared to [^68^Ga]Ga-DOTA-FAPI-46 (1.76 ± 0.53, tissue/muscle ratio), suggesting a slow tumoral clearance. This important finding led to further considerations regarding potential theranostic applications. Moreover, the [^68^Ga]Ga-AAZTA-FAPI-46 tracer uptake magnitude fully agrees with literature data on FAPI-46-based tracers that employed tumour models generated by FAP transfected cell lines (Pang et al. 2023). Indeed, it is worth noting the modest activity injected (1–2 MBq) and the murine model employed (4T1 tumor model) in our study. A large part of literature examples reported indeed a FAP-transfected murine model (Lindner et al. 2018; Loktev et al. 2019; Feng et al. 2024), for which FAP-expression is higher and an activity injected between 2.7 and 8 MBq (Feng et al. 2024; Shang et al. 2024; Ding et al. 2021).

**Fig. 6:**
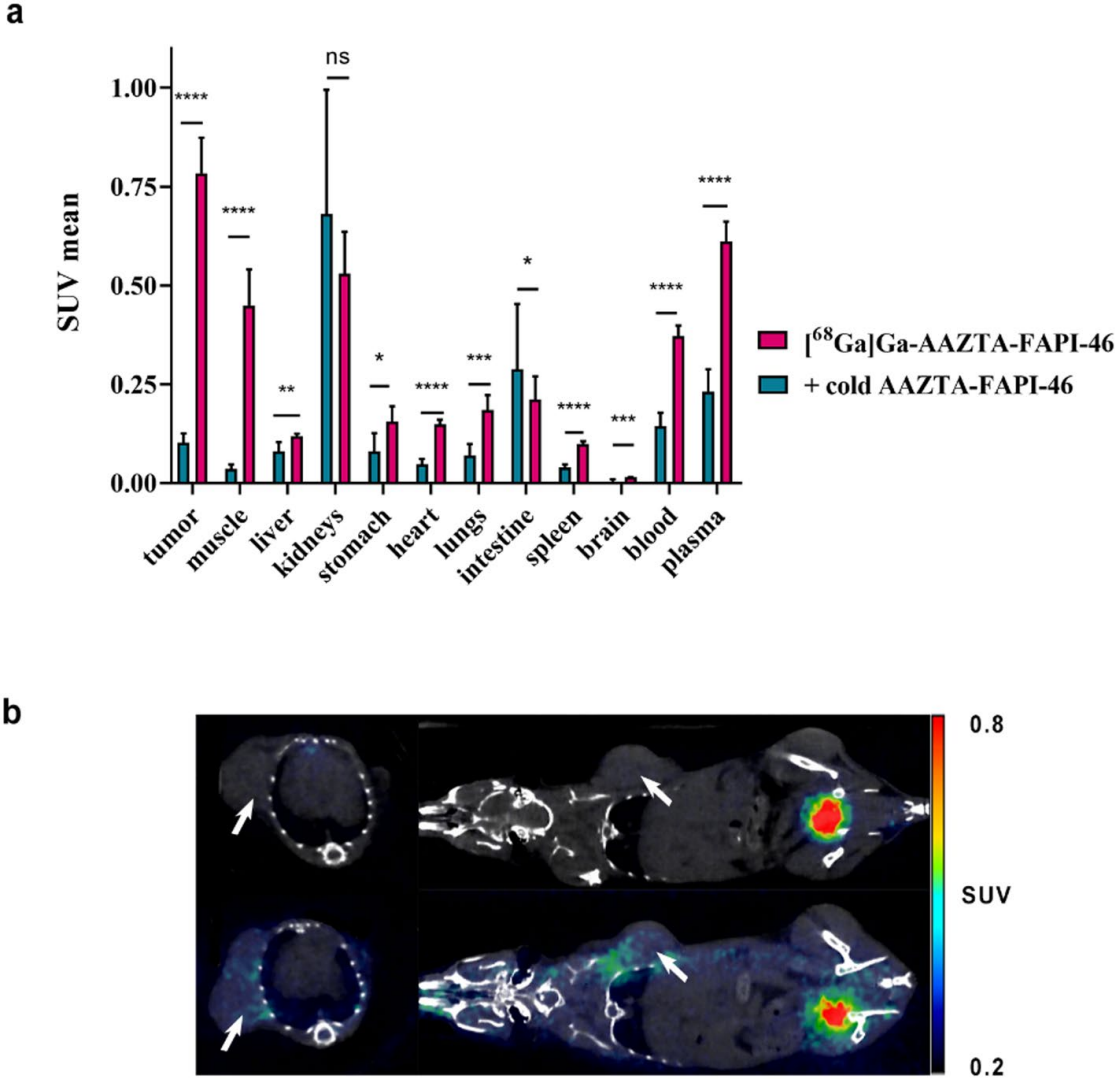
(**a**) Organ uptake 80 min p.i. of ≈ 1.6 MBq [^68^Ga]Ga-AAZTA-FAPI-46 in 4T1-tumor–bearing mice (*n* = 4) in absence (pink) and in presence of 100x-excess of competitor (blue). (**b**) PET/CT images acquired 80 min p.i. of ≈ 1.6 MBq of [^68^Ga]Ga-AAZTA-FAPI-46 in absence (on the bottom) or in presence of 100x-excess of competitor (on the top) in 4T1-tumor-bearing mice. White arrows indicate tumors. PET and CT images are reported in Fig. S7

The overall results of biodistribution experiments were reported in Fig. 6. The tumor uptake was high (0.78 ± 0.09 SUV), especially considering the modest activity injected and the murine model employed (Fig. 6a). 80 min-post-injection the tracer is still circulating as shown from muscle, blood and plasma signal (0.45 ± 0.09 SUV, 0.37 ± 0.03 SUV and 0.61 ± 0.05 SUV respectively). The repartition between the fluid and the corpuscular part of the tracer into the blood was evaluated by discriminating the plasma content. As expected, the main tracer repartition was found into the plasma, likely due to the interaction of the tracer with plasmatic proteins. Regarding the elimination route, 80-minutes p.i. kidneys signal was high as expected (0.53 ± 0.11 SUV), third only to that of tumour. The remaining main organs showed a significantly lower SUV mean values in agreement with previously data on [^68^Ga]Ga-DOTA-FAPI-46 (Loktev et al. 2019).

To proof the in vivo tracer specificity, [^68^Ga]Ga-DOTA-FAPI-46 was injected in presence of 100x-excess of unlabelled tracer, PET/CT images (Fig. 6b - for PET and CT images, refer to **Fig. S7**) were acquired 1 h post-injection and mice were sacrificed 80 min post-injection for biodistribution measurements. The results show the complete drop of the 4T1-tumor-signal (from 0.78 ± 0.09 to 0.10 ± 0.02 SUV) confirming the tracer tumor uptake was FAP-mediated, despite the substitution of the DOTA chelator with the AAZTA chelator. As seen from the Fig. 6 the blocking effect is evident in other peripheral tissues such as muscles, kidneys, lungs, heart and to a lesser extent in the stomach, due to the expression of FAP protein in these tissues as listed in the protein atlas database (proteinatlas.org), and largely reported in other similar studies (Loktev et al. 2019). Inhibition also reduced the concentration of radioactivity in blood and plasma, suggesting that high-dose precursor administration could compete with the binding of the tracer to the plasmatic protein, thus affecting its renal excretion. Furthermore, it can be hypothesized that the large excess of competitor provides a blocking effect even on non-specific binding sites.

## Discussion

A fast and high-radiochemical yield protocol for the radiolabelling of AAZTA-FAPI-46 with gallium-68 was developed, which avoids heating instrumentations and high-temperature conditions. Hence, the translation of the manual protocol to the automated one was facilitated. The major concern about the replacement of DOTA with the AAZTA chelator was related to the eventual modification on the targeting properties of the tracer as well as its in vivo biodistribution. Competition and internalization studies on FAP-expressing cells reveal a FAP specificity in the nanomolar range and a high internalization rate, which was propaedeutic to the preclinical validation of the tracer on a breast cancer murine model. The PET imaging study highlighted the good performance of the tracer with no substantial differences with the DOTA-containing FAPI-46 probe. Tracer uptake measurements following biodistribution experiments finally confirmed and validate the specificity of the [^68^Ga]Ga-AAZTA-FAPI-46 tracer. The results indicated that [^68^Ga]Ga-AAZTA-FAPI-46 exhibited a tumour uptake similar to the DOTA-containing FAPI-46. Moreover, considering the use of a tumour model with natural FAP-expression and the very low activity injected, the significance of the results is further heightened.

## Conclusion

Demonstrating comparable performance with [^68^Ga]Ga-DOTA-FAPI-46, [^68^Ga]Ga-AAZTA-FAPI-46 could provide an alternative option for radiolabelling with gallium-68, with potentially advantageous properties, such as easier radiolabelling protocol, wider application to temperature-sensitive vectors and potential theranostic applications. To further enhance its significance as a tracer, the theranostic performance should be evaluated, including in a future study the use of radionuclides such as lutetium-177.

## Supplementary Information

Below is the link to the electronic supplementary material.

Supplementary Material 1

## Data Availability

The datasets generated during and/or analysed during the current study are available from the corresponding author on reasonable request.
