# Supplementary material for: Preclinical evaluation of [68Ga]Ga-AAZTA-FAPI-46: a novel PET tracer for targeting fibroblast activation protein (FAP)
Source: EJNMMI Radiopharm Chem. 2025 Aug 5;10:51. doi: 10.1186/s41181-025-00375-2 (PMC12325145; doi:10.1186/s41181-025-00375-2)
Supplement: Supplementary file 1 — Supplementary Material 1 [file 41181_2025_375_MOESM1_ESM.docx]

**EJNMMI Radiopharmacy and Chemistry**

Rebecca Rizzo^1^, Paolo Rainone^4,5^, Rachele Stefania^3^, Sara Belloli^4,5^, Silvia Valtorta^4,5^, Angela Coliva^4^, Marco Maspero^4^, Lidia Avalle^3^, Martina Capozza^1^, Rosa Maria Moresco^4,5^, Calogero D’Alessandria^2^, Enzo Terreno^1^

^1^ Center for Biotechnology and Translational Medicine, University of Turin, Piazza Nizza 44/bis, Turin, 10126, Italy

^2^ TU University Hospital rechts der Isar, Department of Nuclear Medicine, , Munich, 81675, Germany

^3^ DISIT, University of Eastern Piedmont, Viale Teresa Michel 11, Alessandria, 15121, Italy

^4^ Nuclear Medicine and PET Cyclotron Unit, IRCCS Ospedale San Raffaele, Milano, Italy

^5^ Institute of Molecular Bioimaging and Physiology-IBFM, CNR, Segrate, Italy

**corresponding author**: enzo.terreno@unito.it

## **Supplementary Information**

**Synthesis of AAZTA-FAPI-46**

**Synthesis of *tert-butyl 6-bromoquinoline-4-carboxylate* (1-ii)**

To a solution of DIC (3.25 ml, 21 mmol, 5.3 eq) in THF (10 ml), *tert*-butanol (2.21 ml, 23.1 mmol, 5.8 eq) and CuI (400 mg, 0.53 mmol, 0.53 eq) were added and the reaction was stirred overnight at room temperature. The suspension was filtrated on celite to eliminate the catalyst and to obtain the *tert-butyl (Z)-N,N'-diisopropylcarbamimidate* (**1**) intermediate. The product was then evaporated *in vacuo* and used without further purification.

To a suspension of *6-bromoquinoline-4-carboxylic acid* (1 g, 3.968 mmol, 1eq) in THF (10 ml), Et_3_N was added (255 µl, 1.83 mmol, 0.46 eq) to get a limpid solution. This solution was treated with the intermediate obtained in the previous step and the mixture was stirred overnight at 50°C. The suspension was centrifugated and the supernatant was concentrated *in vacuo*. The residue was purified by chromatography on silica gel column (DCM:MeOH 100:0🡪 99:0,5🡪99:1) to afford the *tert*-butyl ester (719.4 mg, 2.34 mmol, y: 59%).

UPLC-MS: R_t_= 9.33 min; [M+H]^+^ = 307.97 m/z [M-tBu]^+^ = 252.06 m/z


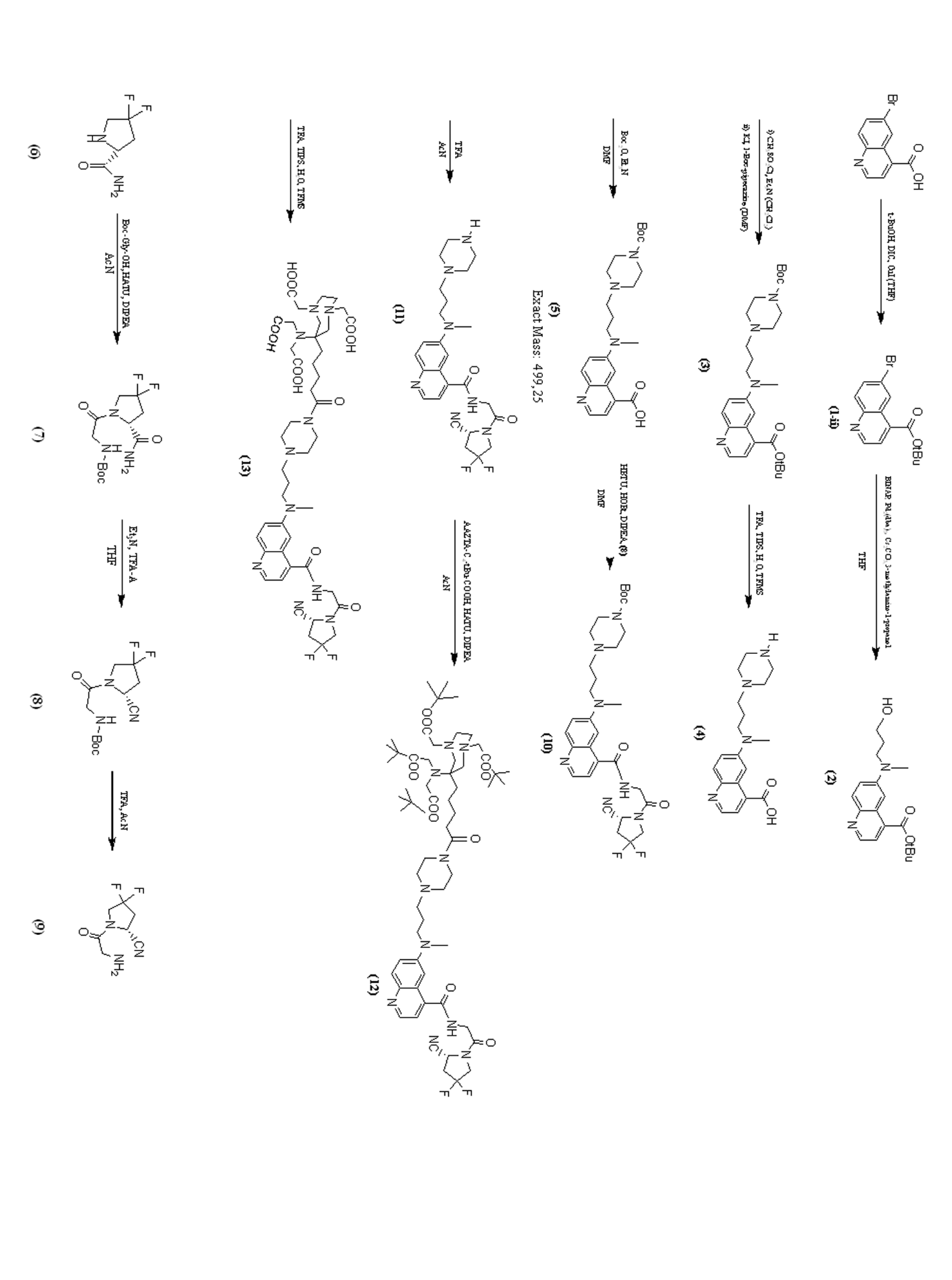


**Fig.S1** Synthetic scheme of AAZTA-FAPI-46

**Synthesis of *tert-butyl 6-((3-hydroxypropyl)(methyl)amino)quinoline-4-carboxylate* (2)**

BINAP (292 mg, 0.468 mmol, 0.2 eq) and Pd_2_(dba)_3_ (215 mg, 0.234 mmol, 0.1 eq) were dissolved in THF (10 ml) and the dark solution was stirred for 10 minutes at 50°C. Then, a solution of *t*-butyl ester intermediate **(1)** (719 mg) in THF (5 ml) was added. The base Cs_2_CO_3_ (4575 mg, 14.04 mmol, 6eq) and 569 µl of 3-methylamino-1-propanol (5.85 mmol, 2.5 eq) were then added. The mixture was stirred under argon atmosphere at 65 °C overnight. The mixture was concentrated *in vacuo* before purifying by chromatography on silica gel column (DCM:MeOH = 95:5) to afford the desired product (403 mg, 1.28 mmol, y: 55%).

UPLC-MS: R_t_= 4.92 min [M+H]^+^ = 317.31 m/z [M-tBu]^+^ = 261.09 m/z

**Synthesis of *tert-butyl 6-((3-(4-(tert-butoxycarbonyl)piperazin-1 yl)propyl)(methyl)amino)quinoline-4-carboxylate* (3)**

The product (**2**) was dissolved in CH_2_Cl_2_ (10ml) and Et_3_N (586 µl, 4.2 mmol, 3.3eq) were added. CH_3_SO_2_Cl (129 µl, 1.66 mmol, 1.3 eq) was dropwise added at 0°C and the mixture was stirred at room temperature. Conversion of the -OH group into the mesylate derivate was monitored by TLC (DMC:MeOH = 95:5) and, after the complete conversion was achieved, the 1-*Boc*-piperazine was added (1.25 g, 7.1 mmol, 5.6 eq). The mixture was stirred for 5 minutes and then the solvent was removed under reduced pressure. The mixture was solubilized into 10 ml of DMF and 315 mg of KI (1.9 mmol, 1.5 eq) were added. The temperature of the reaction was increased up to 60°C and the reaction was stirred overnight. The solvent was removed under vacuum and the resulting mixture was suspended in ethyl acetate (10 ml). Then, the organic layer was washed with water (10 ml) and brine (10 ml), in succession. The organic layer was dried over NaSO_4_ before solvent was removed under vacuum. The residue was purified by chromatography on silica gel column (DCM:MeOH = 98:2) to afford the product (240 mg, 0.496 mmol, y: 39%).

**UPLC-MS** Rt= 5.34 min; m/z: [M+H]^+^ = 485.34 , [M-Boc]^+^ = 385.41

**Synthesis of *6-(methyl(3-(piperazin-1-yl)propyl)amino)quinoline-4-carboxylic acid* (4)**

The product (**3**) was reacted with TFA trifluoroacetic acid (2.2 ml), TIPS triisopropylsilane (60 µl), H_2_0 (60 µl) and TFMSA trifluoromethanesulfonic acid (120 µl) at room temperature for 1 hour or until the reaction was completed as checked-out by TLC (DCM:MeOH = 9:1). After removal of the solvent, the residue was dissolved in diethyl ether to further eliminate TFA residues and to get the precipitation of the red product. The ether was discarded after centrifugation, the product was dissolved into water and lyophilized to afford a red powder (161.1 mg, 0.491 mmol, y: 99%).

**UPLC-MS** Rt=1.01 m/z: [M+H]^+^ = 329.23

**Synthesis of *6-((3-(4-(tert-butoxycarbonyl)piperazin-1-yl)propyl)(methyl)amino)quinoline-4-carboxylic acid* (5)**

The deprotected compound (**4**) was dissolved in DMF (5 ml) and was reacted with Boc_2_O (146 mg, 0.67 mmol, 1.35 eq) and Et_3_N (121 µl, 0.868 mmol,1.75eq) at room temperature for 2h. The reaction progress was monitored by TLC (DCM: MeOH = 9:1) and, once the completion was achieved, the solvent was removed under vacuum. The residue was dissolved in diethyl ether to further eliminate DMF and to afford the precipitation of the red product. Then, by discarding ether after centrifugation, the product was allowed to air dry. The product was used without further purification.

**UPLC-MS** Rt= 3.29 min; m/z [M+H]^+^ = 429.44, [M-Boc]^+^ = 329.23

***Synthesis of tert-butyl (2-(2-carbamoyl-4,4-difluoropyrrolidin-1-yl)-2-oxoethyl) carbamate* (7)**

To a suspension of Boc-Gly-OH (1.033 g, 5.895 mmol, 1.1 eq) in acetonitrile (10 ml), 2.242 g of HATU (5.895 mmol, 1.1 eq) and 3.82 ml of DIPEA (21.436 mmol, 4 eq) were dissolved to get a limpid solution; then, 1 g of *(S)-4,4-difluoropyrrolidine-2-carboxamide hydrochloride* was added and the solution was stirred at room temperature for 3 hours or until TLC (DCM: MeOH = 97:3) showed the reaction was completed. The suspension obtained was centrifuged and the whitish solid was washed twice with DCM. The solid obtained was used without further purification (752 mg, 2.447 mmol, y:46%).

**UPLC-MS** R_t_= 3.60 min; m/z [M+Na]^+^ = 330.02 , [M-Boc]^+^ = 208.19

***Synthesis of tert-butyl (2-(2-cyano-4,4-difluoropyrrolidin-1-yl)-2-oxoethyl) carbamate* (8)**

The previously obtained compound was dissolved into THF (7ml) and 1.7 ml of pyridine (20.8 mmol, 8.5 eq) were added. After leading the temperature to 0°C, a solution of TFA-A trifluoroacetic anhydride (579 µl, 4.16 mmol, 1.7 eq) in THF (3 ml) was dropwise added. The reaction was stirred at 0°C for 90 minutes or until TLC (DCM:MeOH=97:3) showed the conversion of amino group into nitrile group was completed. The solvent was removed under vacuum, the solid was dissolved in ethyl acetate and the organic layer was washed with HCl 2M (3x25ml), a saturated solution of NaHCO_3_ (30ml) and brine (30 ml). The organic layer was dried over NaSO_4_ before removal of the solvent under vacuum (581 mg, 2.01 mmol, y: 82%).

**UPLC-MS** R_t_= 5.01 min; m/z [M+Na]^+^ = 312.05 , [M-Boc]^+^ = 190.08

***Synthesis of 4,4-difluoro-1-glycylpyrrolidine-2-carbonitrile* (9)**

To a suspension of (**8**) in acetonitrile, *p*-toluensulfonic acid (574 mg, 3.015 mmol, 1.5eq) was slowly added. The reaction mixture was stirred at room temperature for 24h and, after removing the solvents under reduced pressure, diethyl ether was added at 0°C to get a whitish solid precipitation. After centrifugation, the supernatant was discarded and the solid was used without further purification (372 mg,1.97 mmol, y: 98%).

**UPLC-MS** R_t_= 1.42 min; m/z [M-Boc]^+^ = 190.08

**Synthesis of *tert-butyl 4-(3-((4-((2-(2-cyano-4,4-difluoropyrrolidin-1-yl)-2-oxoethyl) carbamoyl) quinolin-6-yl)(methyl)amino)propyl)piperazine-1-carboxylate* (10)**

The carboxylic acid compound **(5)** was dissolved in DMF (6 ml) and then HOBt (188mg, 1.39 mmol, 2.8 eq), HBTU (235 mg, 0.62 mmol, 1.25 eq) and DIPEA (111 µl,0.62 mmol, 2.8 eq) were added. The reaction mixture was reacted for 15 minutes before adding a solution of the amine compound **(9)** (135 mg, 0.714 mmol, 1.44 eq) in DMF (2ml). The mixture was reacted for 1 h and, once the completion was achieved, the solvent was removed under vacuum and the product was obtained following the precipitation with diethyl ether, as described before. The solid was then purified by chromatography on silica gel column (DCM:MeOH=9:1) to afford a red solid (281 mg, 0.469 mmol, y: 95%).

**UPLC-MS** Rt= 4.90 min m/z [M+H]^+^ = 600.26 , [M-Boc]^+^ = 500.15

**Synthesis of *4,4-difluoro-1-((2-(6-(methyl(3-(piperazin-1-yl)propyl)amino)quinolin-4-yl)-2-oxoethyl)glycyl)pyrrolidine-2-carbonitrile* (11)**

The previously obtained compound (**10**) was dissolved in acetonitrile (5ml) and reacted with trifluoroacetic acid (10ml) for 1 h, until the deprotection of Boc-group was completed. After reducing solvents under vacuum, the product was precipitated in diethyl ether to afford, after centrifugation, a red powder. The procedure described above was followed. The product was used without further purification (240mg, 0.442 mmol, y: 94 %).

**UPLC-MS** Rt= 0.87 min, m/z [M+H]^+^ = 500.20 [M+2H]^+^ = 250.66

**Synthesis of *AAZTA-C_4_-(tBu)-FAPi-46* (12)**

198 mg of 5-(6-(bis(2-(tert-butoxy)-2-oxoethyl)amino)-1,4-bis(2-(tert-butoxy)-2-oxoethyl)-1,4-diazepan-6-yl)pentanoic acid (0.296 mmol,1.1 eq) were dissolved in 3 ml of ACN and then HATU (113mg, 0.296 mmol, 1.1 eq) and DIPEA (239 µl, 1.34 mmol, 5 eq) were added. After 5 minutes, a solution of compound (**11**) (134 mg, 0.269 mmol, 1 eq) in ACN (2ml) was added to the mixture and the reaction was reacted for 1 hour. After the coupling was completed, the solvent was reduced under vacuum. The mixture was purified by chromatography on silica gel column (DCM:MeOH=9:1) to afford the isolated (**12**) compound (110 mg, 0.0951mmol, y: 35 %).

**UPLC-MS** Rt= 7.78 min [M+H]^+^ = 1153.44 m/z, [M+2H]^+^ = 577.36 m/z

**Synthesis of *AAZTA-C_5_-FAPi-46* (13)**

Finally, the *tert*-butyl groups of AAZTA chelator had to be deprotected. Herein, the compound (**12**) was dissolved in DCM (4 ml) and TFA (4ml) and TIPS (80 µl, 1%) were added. The reaction mixture was shacked for 24 h and, once the total deprotection was achieved, the solvent was removed under reduced pressure. The product was obtained following the precipitation procedure with diethyl ether. After discarding the diethyl ether, the red powder was dissolved in water and lyophilized (81 mg, 0.087 mmol, y: 92%).

**UPLC-MS** Rt= 3.21 min [M+H]^+^ = 929.53 m/z, [M+2H]^+^ = 465.35 m/z

**UPLC-MS analytical method**

Injection volume 5 µl

Flow 0.4 ml/min

| t | % H_2_O (0,1% TFA) | %CH_3_CN |
| --- | --- | --- |
| 0 | 95 | 5 |
| 14 | 0 | 100 |

**HPLC-MS preparative method**

Injection volume 100 µl

[45 mg/ml]

Flow 20 ml/min

| t | % H_2_O (0,1% TFA) | %CH_3_CN |
| --- | --- | --- |
| 0 | 95 | 5 |
| 5 | 95 | 5 |
| 9 | 85 | 15 |
| 16 | 85 | 15 |
| 20 | 40 | 60 |
| 23 | 0 | 100 |

**
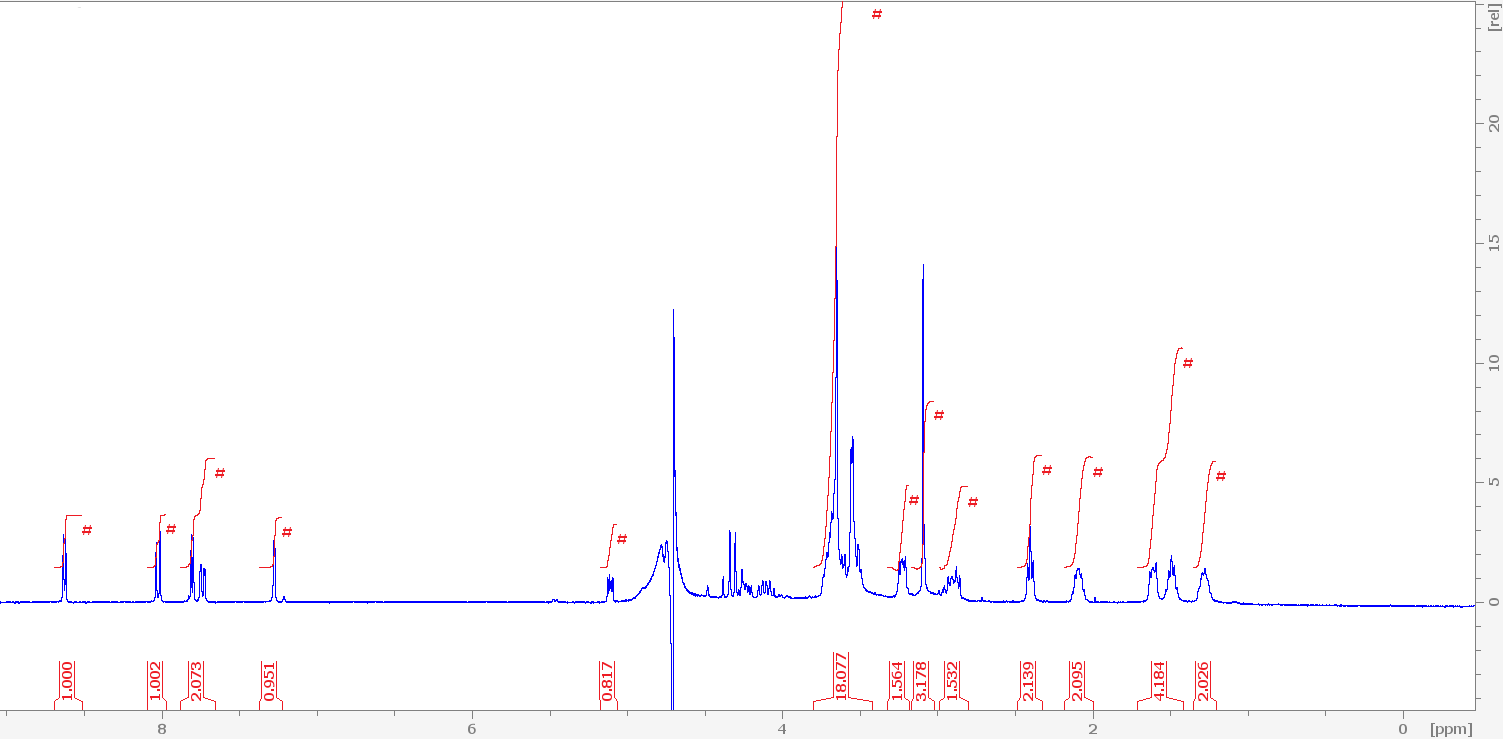
^1^H NMR spectrum**

**
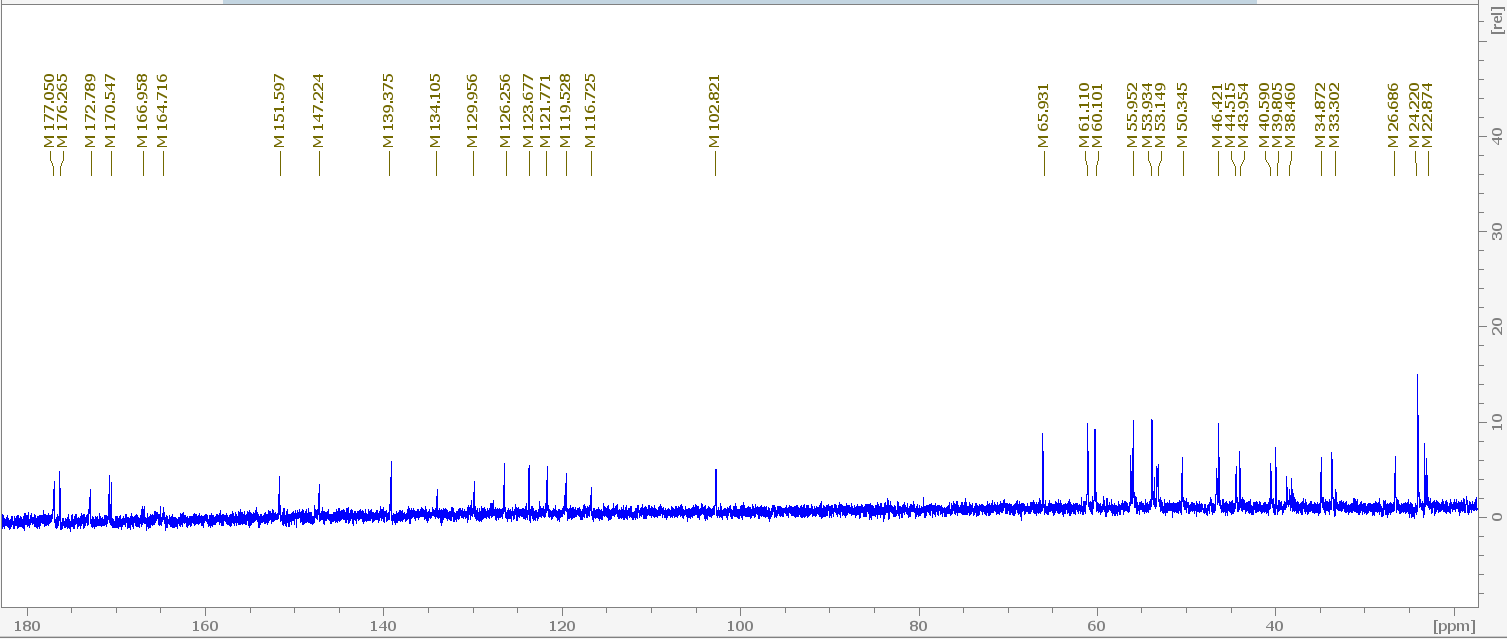
^13^C NMR spectrum**

**Fig.S2** ^1^H-NMR (400 MHz) spectrum of AAZTA-FAPI-46 in D_2_O (T=298K) at the top. Chemicals shift δ (in ppm) and integrations are reported: δ 1.28 (m, 2H), δ 1.49-1.61 (m, 4H), δ 2.09 (t, 2H), δ 2.40 (t, 2H), δ 2.89 (m, 2H), δ 3.09 (s, 3H), δ 3.23 (m, 2H), δ 3.54 (18H), δ 5.11 (m, 1H), δ 7.27 (d, 1H), δ 7.73 (d, 1H), δ 7.80 (d, 1H), δ 8.01 (d, 1H), δ 8.62 (d, 1H). Peak at 4.70 ppm is referred to water suppressed signal. Carboxylic and amidic protons are not visible because of the exchange with solvent.

^13^C-NMR (400 MHz) spectrum of AAZTA-FAPI-46 in D_2_O (T=298K) at the bottom. Chemicals shift δ (in ppm): δ 177-170 ppm referred to carboxylic carbons, δ 166-164 ppm referred to amidic carbons, δ 151-102 ppm referred to quinoline carbons, 102, 53, 35, 33 ppm referred to pyrrolidine carbons, 55 and 50 ppm referred to AAZTA cycle’s carbons, 22-60 ppm referred to aliphatic carbons.

**b**


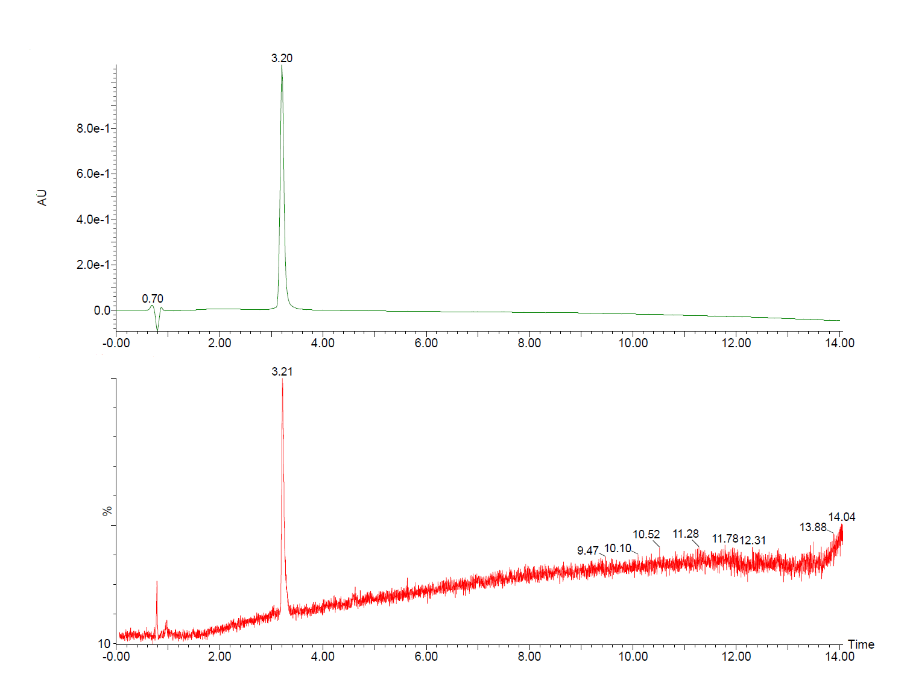

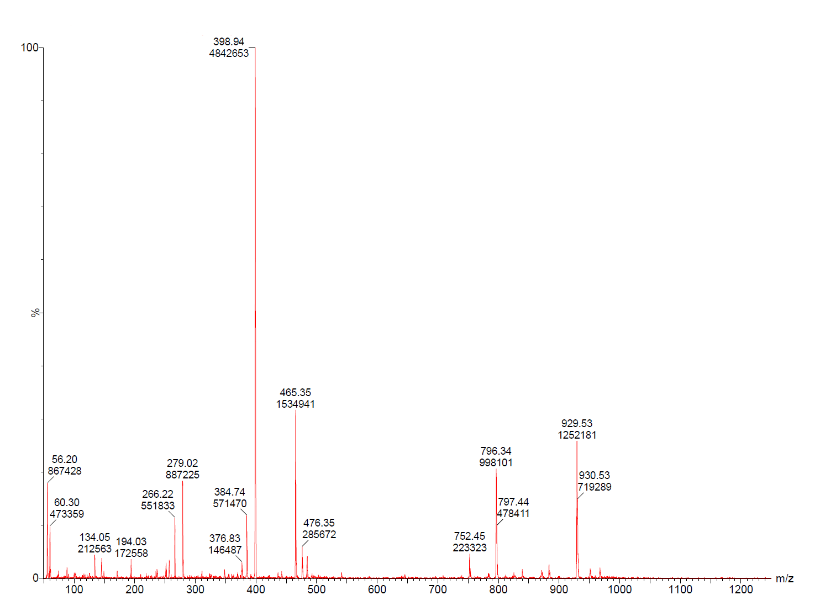


**a**

**Fig.S3** (a) Chromatogram acquired at 254 nm of purified AAZTA-FAPI-46 on Acquity® UPLC BEH C18 column. (b) ESI(+) mass spectrum of AAZTA-FAPI-46

**Buffers for radiolabeling procedure**

HEPES 0,4M for pH=3.5; NaOAc 0.4M for pH ranging from 4 to 5.

**Fig.S4** ^68^Ga-incorporation (sum of labelled AAZTA-FAPI-46 and colloidal contribute) at different pH values (RT, 15 minutes, 40 µM) assessed by radio-TLC


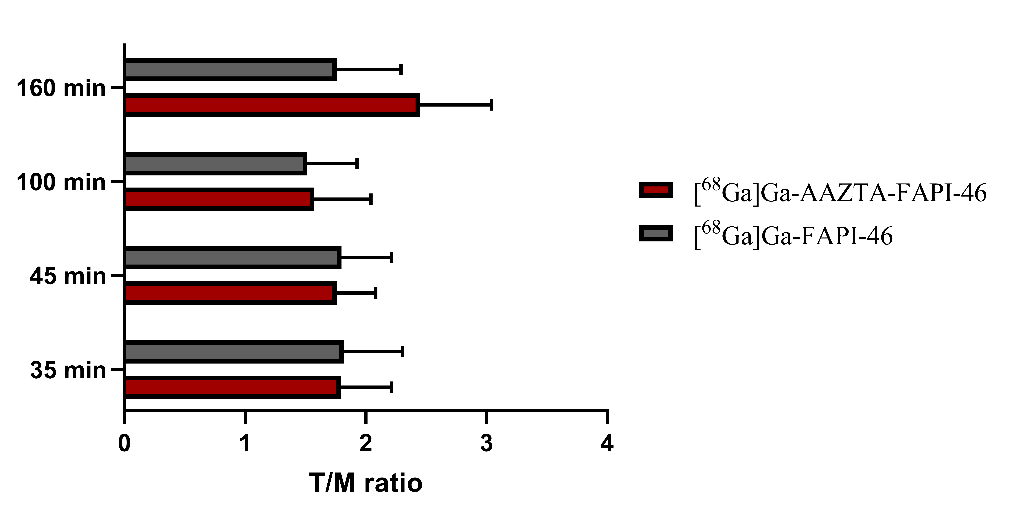


**Fig.S5** Tissue/muscle ratio values found from PET/CT images acquired after *i.v.* administration of ≈ 1.9 MBq of [^68^Ga]Ga-AAZTA-FAPI-46 (n=8) or [^68^Ga]Ga-DOTA-FAPI-46 (n=8).

**t-test: organs uptake competition *in vivo* study.**

tumor *** 0.0004

muscle ** 0.0057

liver ns 0.2132

kidneys ns 0.9951

stomach ns 0.2536

heart **** <0.0001

lungs * 0.0106

intestine ns 0.9963

spleen **** <0.0001

brain ns 0.1285

blood **** <0.0001

plasma **** <0.0001

**
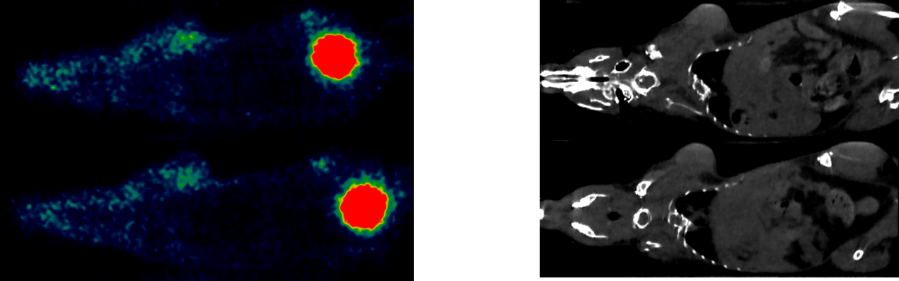
**

**Fig. S6** PET images on the left and CT images on the right relative to PET/CT images in Fig. 5.

**
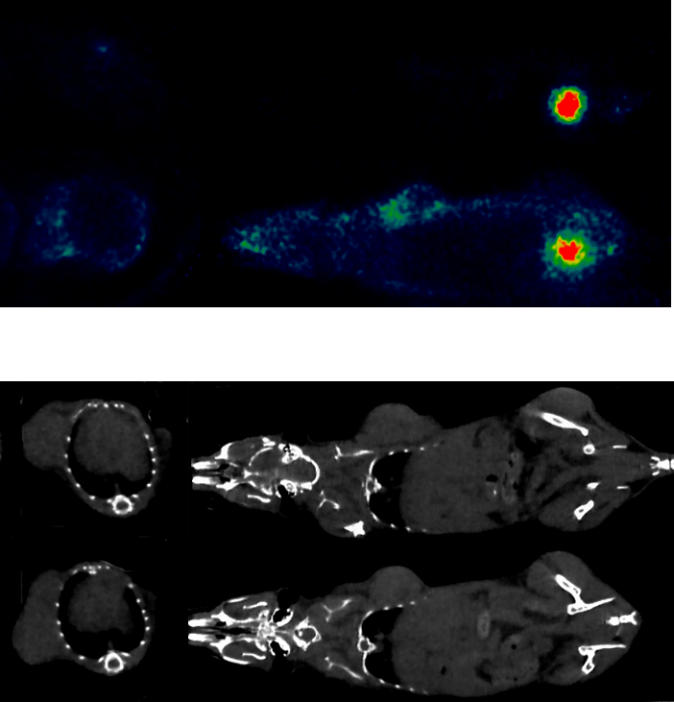
**

**Fig. S7** PET images on the top and CT images on the bottom relative to PET/CT images in Fig. 6.
